# Fast Kinematic Re-Calibration for Industrial Robot Arms

**DOI:** 10.3390/s22062295

**Published:** 2022-03-16

**Authors:** Sreekanth Kana, Juhi Gurnani, Vishal Ramanathan, Sri Harsha Turlapati, Mohammad Zaidi Ariffin, Domenico Campolo

**Affiliations:** School of Mechanical and Aerospace Engineering, Nanyang Technological University, Singapore 639798, Singapore; sreekanth.kana@ntu.edu.sg (S.K.); jgurnani@ntu.edu.sg (J.G.); vishal.pr@ntu.edu.sg (V.R.); turl0001@e.ntu.edu.sg (S.H.T.); mohd.zaidi@ntu.edu.sg (M.Z.A.)

**Keywords:** robot calibration, kinematic re-calibration, positional accuracy, kinematic modelling, linear regression, parameter identification, industrial robots

## Abstract

Accurate kinematic modelling is pivotal in the safe and reliable execution of both contact and non-contact robotic applications. The kinematic models provided by robot manufacturers are valid only under ideal conditions and it is necessary to account for the manufacturing errors, particularly the joint offsets introduced during the assembling stages, which is identified as the underlying problem for position inaccuracy in more than 90% of the situations. This work was motivated by a very practical need, namely the discrepancy in terms of end-effector kinematics as computed by factory-calibrated internal controller and the nominal kinematic model as per robot datasheet. Even though the problem of robot calibration is not new, the focus is generally on the deployment of external measurement devices (for open loop calibration) or mechanical fixtures (for closed loop calibration). On the other hand, we use the factory-calibrated controller as an ‘oracle’ for our fast-recalibration approach. This allows extracting calibrated intrinsic parameters (e.g., link lengths) otherwise not directly available from the ‘oracle’, for use in ad-hoc control strategies. In this process, we minimize the kinematic mismatch between the ideal and the factory-calibrated robot models for a Kinova Gen3 ultra-lightweight robot by compensating for the joint zero position error and the possible variations in the link lengths. Experimental analysis has been presented to validate the proposed method, followed by the error comparison between the calibrated and un-calibrated models over training and test sets.

## 1. Introduction

Robots are being widely adopted on the industrial floor nowadays, with the aim to automate more and more manufacturing processes for increased efficiency and production. In addition, in industrial robotic applications, position accuracy and repeatability are the most fundamental attributes for automating flexible manufacturing/assembly tasks [1]. When operating robots in position control mode to trace a mathematically described trajectory, repeatability alone is not sufficient for it to trace that path. There arises a need to measure how accurately the robot moves along the generated path [2].

Positional accuracy can be defined as the difference between the position of a commanded pose and the barycentre of the attained position [3]. Most manufacturing processes involve low tolerances between various components, thereby requiring high positional accuracy. Errors higher than a couple of millimetres may result in wear or damage to the parts/objects involved. In such scenarios, accuracy can be seen as an important indicator of performance. Other examples of robotic applications that require high absolute positioning accuracy include offline programming, visual servoing and laser cutting [4].

Inaccuracies in robots are caused by several factors and in particular, these sources of error can be divided into geometric and non-geometric factors. The most common sources are geometric in nature such as minor axis misalignments from the model which arise during production, errors in joint positions and joint angles [5]. Robots are usually manufactured to their specifications at a minimum tolerance to ensure highly precise geometric configuration of the joint axes and transmission mechanisms for actuating the joints. However, oftentimes after assembly of all the parts, it becomes challenging to accurately measure these specifications and this can result in some deviations from the model. Apart from the manufacturing errors, robots operating in flexible industrial assembly systems also have abrasive wearing of transmission parts due to very low tolerances [6]. These may even require repairs to be conducted at times which can further result in errors with respect to the actual model and in order to compensate for these errors, a re-calibration of the robot may be needed [7].

The non-geometric factors contributing to accuracy errors consist of structural deformations such as backlash [8] and clearance in the transmission system as well as link flexibility, joint flexibility, slip-stick phenomena and thermal expansion [5]. However, these errors are considered to be much smaller compared to those originating from geometric factors [7,9]. Therefore, having a reliable method for calibrating robots is essential to ensure position accuracy at all times.

The kinematic calibration technique presented in this paper was motivated by a very practical need, namely the discrepancy in terms of end-effector kinematics as computed by factory-calibrated internal controller and the nominal kinematic model as per robot datasheet. The proposed approach focuses on the static open-loop re-calibration of the Kinova Gen3 robot arm; however, the procedure is generic enough to be applied to other serial manipulators.

Even though the problem of robot calibration is not something new, most of the existing methodologies require external measuring instruments, such as laser trackers or some kind of mechanical fixtures. This not only makes the calibration a laborious procedure in terms of the experimental setup and establishing data synchronization across multiples devices but also introduces challenges under limited robotic workspace (such as factory floor).

While many of the methods address the calibration problem by introducing corrections in the final end-effector pose, our approach focuses on correcting the kinematic model itself (i.e., in terms of the joint and the link offsets). As a result, the differentials (and hence the Jacobian) are easily obtained, which otherwise is not available from the factory calibrated feedback. Calibrated models are essential in the dynamic control of robots, where the computation of both forward and inverse dynamics of the robot depends heavily on the underlying model.

Hence, the contribution of our work can be summarized as (i) a fast calibration approach without the use of external measuring devices or mechanical fixtures but simply utilizing the factory calibrated kinematics as the ground truth and (ii) a way to calibrate the kinematic robot model itself rather than just the corrected end-effector pose.

The rest of the paper is organized as follows. In Section 2, we discuss the relevant robot calibration approaches followed by Section 3, where the kinematic modelling for the robot is reviewed. In Section 4, the methodology for calibration is discussed. Section 5 outlines the experimental validation of the proposed approach followed by the discussion and conclusion in Section 7 and Section 8 respectively.

## 2. Existing Methodologies for Kinematic Calibration

Robot calibration is performed to improve the positional accuracy of the end-effector by accurately calibrating the kinematic parameters, which are proven to be major contributors of the positioning error [6,10]. Kinematic calibration of robots can be done in two different ways namely: model-based and model-less. The model-based methods are more commonly used for robot calibration [6]. However, some studies have also implemented model-less calibration techniques such as [11,12,13,14]. Such techniques involve building relation between the position errors of robots and workspace or joint space [6].

The model-based calibration procedure in general involves developing a model whose parameters accurately represent those of the actual robot then accurately measuring specific features of the real robot followed by the computation of the parameter values for which the model reflects the measurements made [7]. Kinematic or Level 2 calibration is known to improve the robot accuracy across the entire volume of its configuration space. In the model-based approach of kinematic calibration, the geometric factors are applied to identify model parameters. Non-geometrical error sources can be minimized through Level 3 calibration. However, since these non-geometrical errors account for a rather small percentage of the total error, consideration of geometrical errors alone is usually sufficient for a simplified calibration model as they account for a considerable proportion of the end-effector pose error [7,15]. Therefore, in this study we propose a quick re-calibration (Level 2) procedure for serial robot arms by using the geometric factors for parameter identification. Non-geometric calibration is out of the scope of the current work.

Kinematic calibration is usually carried out in following four steps [4,6,16]: (i) Modeling, (ii) Measurement, (iii) Identification and (iv) Compensation/Correction. Before the measurements can be made, the kinematic error model for the robot must be transformed into an identification model. The identification model is a representation of the mapping from pose errors of the end-effector to the unknown geometric errors. The actual values of pose errors are then measured for different configurations using measurement devices and then input to the identification model wherein the geometric errors are computed using numerical methods before being compensated for using hardware/software [17].

Several studies have been conducted so far on the kinematic calibration of industrial robots. Denavit–Hartenberg (DH) modelling is the most popular kinematic modelling technique for serial robot arms; however, it is unable to address the singularity issue of two adjacent parallel joints. Ref. [10] presents a modified DH model (MDH) by introducing a new rotation parameter to overcome this difficulty. Many other works have dealt with the singularity problem through the use of Product-of-Exponentials (POE) based modelling [18,19,20].

Most calibration methods compare the taught point positions of a robot with measurements relating the end-effector to an external 3D measuring device such as a laser tracker [21,22,23,24,25,26], theodolite measurement devices [27] or coordinate measuring machines (CMM) [28,29]. These are known as open-loop calibration techniques [30]. Based on the measurements made, the kinematic parameters of the mathematical model of the robot are then corrected to minimize the difference between the positions where the robot thinks it is and its actual position in the workspace [1]. Ref. [28] performed online pose measurement with an optical CMM and used that as a feedback to steer the end-effector of the robot accurately, to the desired pose. Their approach increased positional accuracy of a robot independent of its kinematic parameters. Ref. [31] presented a method for calibration using distance and sphere constraints to improve robot accuracy in a specific workspace. Spheres with precisely known distances from each other were probed by the robot end-effector multiple times and the measured pose values were then compared with the poses calculated from the kinematic model in an iterative process until the root mean square (RMS) error between the iterations dropped below the specified threshold. Ref. [32] calibrated the robot parameters by controlling six-axis industrial robot arms to get to the same location in different poses. Different identification and compensation methods were proposed that could be mixed and matched to obtain optimal solutions depending on the operational environment.

Table 1 reviews model-based calibration techniques implemented in some of the recent works conducted on kinematic calibration as well as the proposed method for comparison. It can be observed that most of these techniques cater to the experimental environment, requiring external measuring instruments such as laser trackers. This makes it a very time consuming procedure which is only suitable for laboratory environments and not for industrial settings such as automated assembly lines [16]. Thus, to address the research gap on fast calibration methods, this work focuses on fast open-loop re-calibration. The ground truth for measurement of the end-effector pose was not an external measuring device but instead the kinematic feedback provided by the robot controller into which the calibration parameters are implicitly modelled. This calibrated forward kinematics (FK) model was not explicitly available to us; however, the feedback was considered accurate and further used to validate our experiments. A robot kinematic model is characterized by a non-linear function that relates link geometric parameters and joint variables to the robot end-effector pose [5]. To fit this model to experimental data, it needs to be linearized and solved. For parameter identification, different authors have so far implemented different methods of linearizing the kinematic models of their robots. Ref. [33] used least-square minimization (LSM) and single value deposition (SVD) to calibrate parameter errors. Ref. [34] also performed the parameter identification procedure by solving a linearized least square problem for which a ballbar measurement device was used. Linear least square algorithms are usually applied owing to their quick convergence rates [7].

Even though the problem of robot calibration is widely explored, the focus is generally on the deployment of external measurement devices (for open loop calibration [9,28]) or mechanical fixtures (for closed loop calibration [35,36]). In the approach presented in this paper, a model-based fast-kinematic re-calibration is devised whereby we use the factory-calibrated controller as an ‘oracle’ for our fast-recalibration approach.This allows for the extraction of calibrated intrinsic parameters (e.g., link lengths) otherwise not directly available from the ‘oracle’, for use in ad-hoc control strategies. Here, we account for the small variations in both the zero joint offset and the link offsets by minimizing the mismatch between the nominal kinematic model as per robot datasheet and the ‘oracle’. While the problem can be formulated mathematically as a non-linear regression problem, by making the assumption that the end-effector orientation is unaffected by the variations in the link dimensions, it is possible to solve for the above offsets by solving two separate linear regression problems, which simplifies the computation. Concretely, we proceed by first solving for the zero joint offsets and then identifying the link offsets.

## 3. Kinematic Modelling

The first step towards a typical robot calibration is the modelling of the end-effector/tool pose (i.e., the forward kinematics) with respect to a reference frame, which in general is the robot base frame itself. This can be done in several ways, of which the 4-parameter DH (Denavit–Hatenberg) convention is most widely employed. In this paper, the robot platform used for experimental validation is the Kinova Gen3 ultra-lightweight robot arm and hence we employ the DH representation in order to adhere to the convention followed by the datasheet in building the ideal forward kinematic model.

For a robot manipulator with its *N* joints assuming the configuration $q\in R^{N}$, the forward kinematics can be expressed as a homogeneous transformation between the end-effector and base coordinate systems of the robot as [38]

$$\mathrm{FK}\left(q\right)=T_{1}^{B}\left(q_{1}\right)\;T_{2}^{1}\left(q_{2}\right)\ldots T_{E}^{N}\left(q_{n}\right)=\left[\begin{array}{cc} R(q) & P(q)\\ 0 & 1 \end{array}\right],$$

where $R\left(q\right)\in R^{3\times 3}$ and $P\left(q\right)\in R^{3\times 1}$ represent the end-effector orientation and position with respect to the robot base frame,

$$\mathrm{and}\;\;T_{n}^{n-1}=\left[\begin{array}{cc} R(q_{n}) & p_{n}\\ 0 & 1 \end{array}\right]$$

Here, B,E respectively represent the robot base frame and the end-effector frame. $R(q_{n})$ and $p_{n}$ represent the rotation and the translation between the (n−1)th and the nth frame respectively. Note that for the nth joint, the homogeneous transformation $T_{n}^{n-1}$ depends only on the joint angle $q_{n}$.

We can now define the body Jacobian matrix for the manipulator as [38,39]

$$J_{body}=\left[{\mathrm{Ad}}_{T_{E}^{1}}^{-1}\xi_{b1},\;{\mathrm{Ad}}_{T_{E}^{2}}^{-1}\xi_{b2},\ldots ,{\mathrm{Ad}}_{T_{E}^{7}}^{-1}\xi_{b7}\right],$$

where $\xi_{bn}={\left(T_{n}^{n-1}\right)}^{-1}\cdot \partial_{q_{n}}T_{n}^{n-1}$ and ${\mathrm{Ad}}_{T_{E}^{n}}$ represent the adjoint matrix (The adjoint of a homogeneous transformation matrix $T=\left[\begin{array}{cc} R & p\\ 0 & 1 \end{array}\right]$ is obtained as ${\mathrm{Ad}}_{T}=\left[\begin{array}{cc} R & 0\\ \hat{p}R & R \end{array}\right]$, where $\hat{.}$ is used to denote the matrix representation) for the homogenous transformation of the end-effector frame to the nth joint frame.

The body Jacobian can be re-written as

$$J_{body}=\left[\begin{array}{c} J_{pos}\\ J_{rot} \end{array}\right],$$

where $J_{pos}$ and $J_{rot}\in R^{3\times N}$ are, respectively, the top and bottom submatrices.

**Figure 1:**
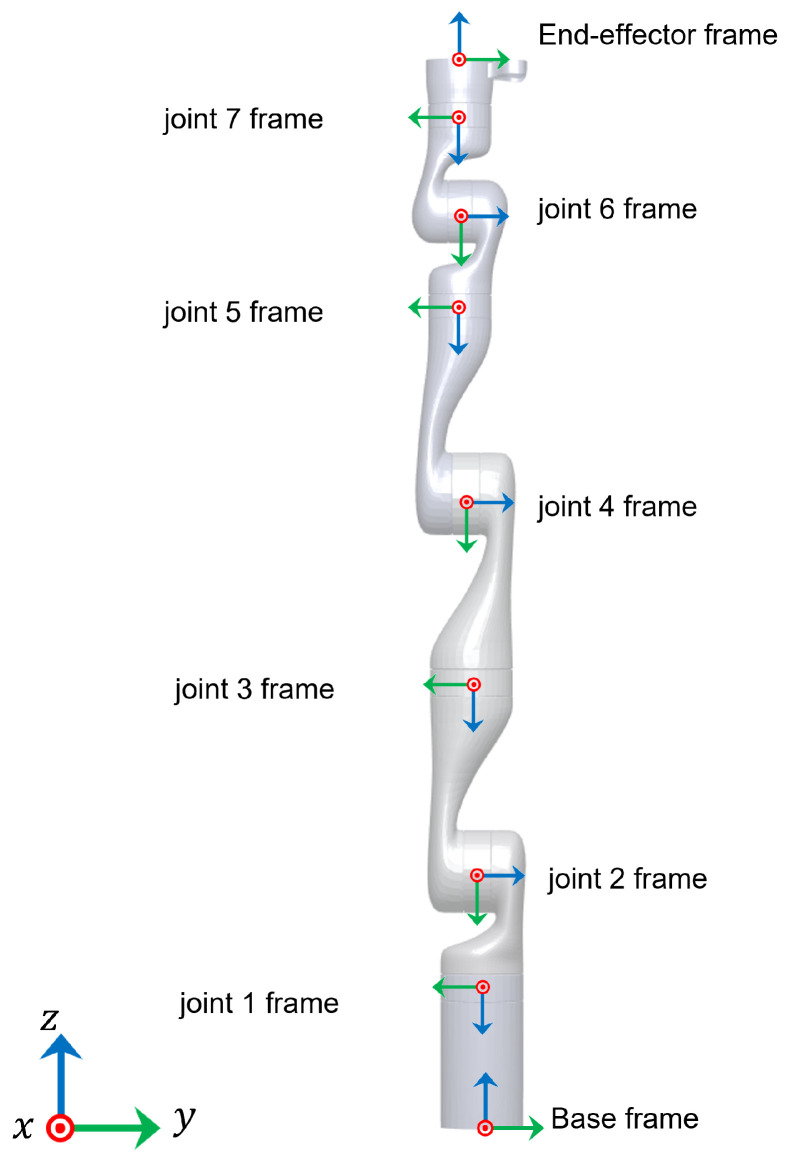
Kinova Gen3 joint frames at zero configuration.

With reference to Figure 1, the homogeneous transformation matrices between the joint frames for a Kinova Gen3 ultra-lightweight robot at zero configuration are as shown in Table 2.

## 4. Parameter Identification and Compensation

The kinematic model available from the manufacturer (for example, the one shown in Table 2) is ideal and does not represent the actual kinematics of the robot after the manufacturing and the assembling stages. In this section, we perform a fast kinematic calibration to account for this discrepancy.

To perform the calibration, we consider a Kinova Gen3 ultra-lightweight robot with 7 degrees of freedom, with no end-effector mounting. However, the methodology is general enough to be adapted for other industrial robots with arbitrary number of degrees of freedom.

Our approach is based on the assumption that the actual forwards kinematics measurements are known. While in some cases, the calibrated parameters are incorporated implicitly in the robot controller (e.g., Kinova Gen3) to provide an accurate feedback of the tool pose, in other instances, one can always make use of external measurement systems calibrated to the robot base frame to identify the actual forward kinematics. In our approach to facilitate a fast kinematic calibration, instead of using an external measuring device to set a ground truth for the tool pose, we consider the feedback tool pose from the robot controller (which is factory calibrated) itself as the ground truth.

For a *N*-joint serial manipulator, consider the forward kinematics as given by the uncorrected (i.e., the ideal) model from Equation (3)

(1) $${\mathrm{FK}}_{p}\left(q\right)=\left[\begin{array}{cc} R(q) & P_{p}\left(q\right)\\ 0 & 1 \end{array}\right]$$

(Note: The subscript p is to show that the forward kinematics is defined for nominal geometric (link) lengths, possibly from CAD models).

However, as discussed in the previous section, the specified transformations hold true only under ideal conditions as variations are introduced during manufacturing and assembling stages. In this paper, we compensate for the zero joint offsets (which henceforth are called *angular offsets* ($\delta q=[q_{1},\ldots ,q_{N}])$ generated in the assembly stage and also for possible deviations of the link dimensions (*linear offsets* ($\delta p=[\delta p_{1},\ldots ,\delta p_{N+1}],$ where $\delta p_{n}\in R^{3\times 1}$)) from their CAD models due to the manufacturing errors. To account for these variations, the transformation between two joint frames can be re-defined as

(2) $$T_{n}^{n-1}=\left[\begin{array}{cc} R(q_{n}+\delta q_{n}) & p_{n}+\delta p_{n}\\ 0 & 1 \end{array}\right],$$

where $p_{n}\in R^{3\times 1}$ is the position vector (say, given by the CAD model) of joint frame *n* with respect to joint frame (n−1) and $\delta p_{n}\in R^{3\times 1}$ is the vector of linear offsets for the (n−1)th link.

Equation (2) on substituting in Equation (3) gives us the corrected forward kinematics which can be denoted as

(3) $${\mathrm{FK}}_{p+\delta p}\left(q+\delta q\right)=\left[\begin{array}{cc} R(q+\delta q) & P_{p+\delta p}\left(q+\delta q\right)\\ 0 & 1 \end{array}\right]$$

Assuming that the factory calibrated tool pose available as a feedback from the controller is a sufficiently accurate representation of the actual forward kinematics, the ground truth can be established as

$$\tilde{\mathrm{FK}}\left(q\right)=\left[\begin{array}{cc} \tilde{R}\left(q\right) & \tilde{P}\left(q\right)\\ 0 & 1 \end{array}\right]$$

Hence, our goal is to identify the small angular ($\delta q^{*}$) and linear ($\delta p^{*}$) adjustments to minimize, in some sense, the difference between $\tilde{\mathrm{FK}}\left(q\right)$ and ${\mathrm{FK}}_{p+\delta p}\left(q+\delta q\right)$.

### 4.1. Identification of Angular Offsets ($\delta q^{*})$

The problem of calibration require us to compensate for both the linear and angular offset which results in a non-linear regression problem. However, for small enough angular offsets, under the assumption that the task-space orientation of the robot is dependent only on the angular offsets, the non-linear regression problem can be turned into two separate linear regression problems for determining the linear and the angular offsets as outlined as follows.

Let R(q+δq) be the rotation matrix with joint offset correction and $\tilde{R}\left(q\right)$ be the known rotation matrix.

At a first order, the vicinity of R(q+δq) and $\tilde{R}\left(q\right)$ can be expressed as

$$R\left(q+\delta q\right)\approx \tilde{R}\left(q\right)$$

$$R\left(q\right)+\frac{\partial R(q)}{\partial q}\;\delta q\approx \tilde{R}\left(q\right)$$

$$I_{3}+R^{T}\frac{\partial R}{\partial q}\;\delta q\approx R^{T}\tilde{R}$$

(4) $$R^{T}\frac{\partial R}{\partial q}\;\delta q\approx R^{T}\tilde{R}-I_{3}$$

Recall that the Cartesian angular velocity $\omega =J_{rot}\dot{q}$ and that $\hat{\omega }=R^{T}\partial_{q}R\;\dot{q}$, therefore

(5) $$J_{rot}\dot{q}={\left(R^{T}\partial_{q}R\dot{q}\right)}^{\vee }$$

Hence Equation (4) can be re-written as

$$J_{rot}\delta q\approx {\left(R^{T}\tilde{R}-I_{3}\right)}^{\vee },$$

where the ${\left(\cdot \right)}^{\vee }$ operator turns 3×3 skew-symmetric matrices into corresponding 3D vectors.

Given a number of *M* robot joint configurations $q_{m}\in R^{N}$, m=1,…,M, we determine an optimal estimate $\delta q^{*}\in R^{N}$, via linear regression for the following system

(6) $$\left[\begin{array}{c} J_{rot}\left(q_{1}\right)\\ \vdots \\ J_{rot}\left(q_{m}\right) \end{array}\right]\delta q=\left[\begin{array}{c} {\left(R_{1}^{T}{\tilde{R}}_{1}-I_{3}\right)}^{\vee }\\ \vdots \\ {\left(R_{M}^{T}{\tilde{R}}_{M}-I_{3}\right)}^{\vee } \end{array}\right],$$

where $R_{m}\equiv R\left(q_{m}\right)$ and ${\tilde{R}}_{m}\equiv \tilde{R}\left(q_{m}\right)$

### 4.2. Identification of Linear Offsets ($\delta p^{*})$

Once the calibration for the joint offset is done, the angular adjustments can be taken into account by rewriting

$$q^{\prime }:=q+\delta q^{*}$$

Now, the linear offsets δp are accommodated into the robot kinematics by re-writing the homogeneous transformation between the joint frames as

(7) $${\mathrm{FK}}_{\left(p+\delta p\right)}\left(q^{\prime }\right)=\left[\begin{array}{cc} R(q^{\prime }) & P_{\left(p+\delta p\right)}\left(q^{\prime }\right)\\ 0 & 1 \end{array}\right]$$

For the same number of *M* measurements, we find the linear offsets δp that minimize the difference

(8) $$P_{p+\delta p}\left(q_{m}^{\prime }\right)-\tilde{P}\left(q_{m}\right),$$

where $P_{p+\delta p}$ is the corrected end-effector position and $\tilde{P}\left(q_{i}\right)$ is the known robot position.

The first order approximation of the error can be written as

(9) $$P_{p+\delta p}\left(q_{m}^{\prime }\right)-\tilde{P}\left(q_{m}\right)\approx P_{p}\left(q_{m}^{\prime }\right)-\tilde{P}\left(q_{m}\right)+\nabla_{p}P\;\delta p$$

Therefore, the optimal optimal estimate $\delta p^{*}$ can be computed as a linear regression of the following (linear) system

(10) $$\left[\begin{array}{c} \nabla_{p}P\left(q_{1}^{\prime }\right)\\ \vdots \\ \nabla_{p}P\left(q_{M}^{\prime }\right) \end{array}\right]\delta p=\left[\begin{array}{c} \tilde{P}\left(q_{1}\right)-P_{p}\left(q_{1}^{\prime }\right)\\ \vdots \\ \tilde{P}\left(q_{M}\right)-P_{p}\left(q_{M}^{\prime }\right) \end{array}\right]$$

The solution to the above equation gives us the linear adjustments to be made, which an be taken into account as

$$p^{\prime }:=p+\delta p^{*}$$

Hence, for a given joint configuration $q\in R^{N}$, the calibrated forward kinematic model can be represented as

(11) $${\mathrm{FK}}_{p^{\prime }}\left(q^{\prime }\right)=\left[\begin{array}{cc} R(q^{\prime }) & P_{p^{\prime }}\left(q^{\prime }\right)\\ 0 & 1 \end{array}\right]$$

## 5. Experimental Validation

### 5.1. Parameter Identification from the Training Dataset

**Figure 2:**
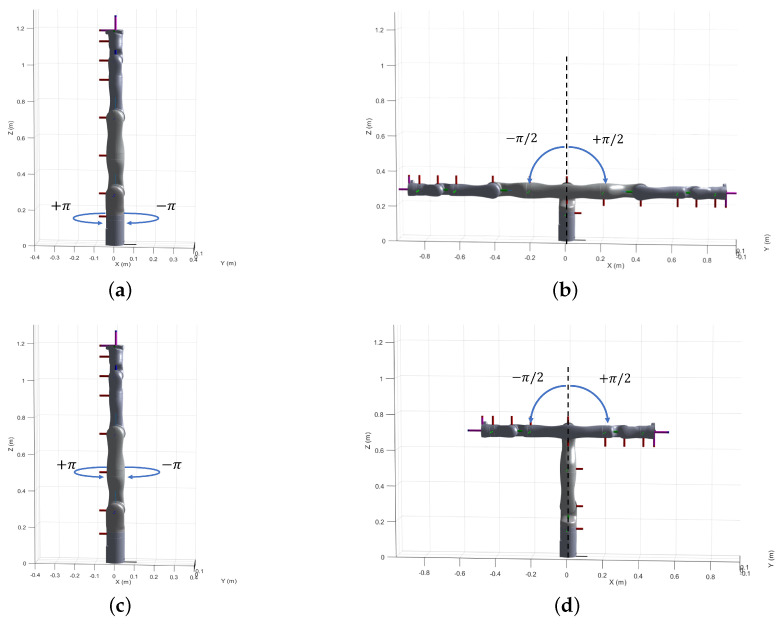

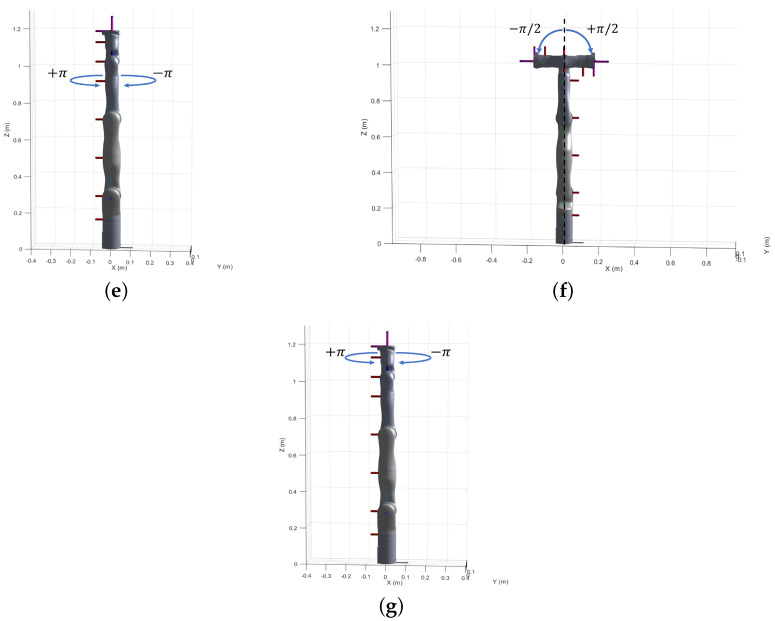
Commanded robot joint motion to generate the training dataset. (**a**) Joint 1. (**b**) Joint 2. (**c**) Joint 3. (**d**) Joint 4. (**e**) Joint 5. (**f**) Joint 6. (**g**) Joint 7.

To validate the proposed approach, we collected a set of training data samples by executing the robot motion in the following manner. Joints with odd indices were commanded to move from 0 to +π rad followed by a motion from 0 to −π rad and the joints with even indices were moved from 0 to $\frac{\pi }{2}$ rad followed by a motion from 0 to $-\frac{\pi }{2}$ rad (due to self-collision constraints). Each joint was moved independently while the rest of the joints were set at the respective zero positions, i.e., for the *i*th joint, $q_{j}=0\;\forall j:j\neq i$ (See Figure 2). The end-effector positions and orientations (i.e., the feedback from the controller) along with the joint configurations were logged during the robot motion. Once the samples were collected, the linear and the angular offsets were identified (Table 3) respectively with the help of Equations (6) and (10) to obtain the corrected kinematic model, which we denote as Model 1.

### 5.2. Validation on the Training Dataset

**Figure 3:**
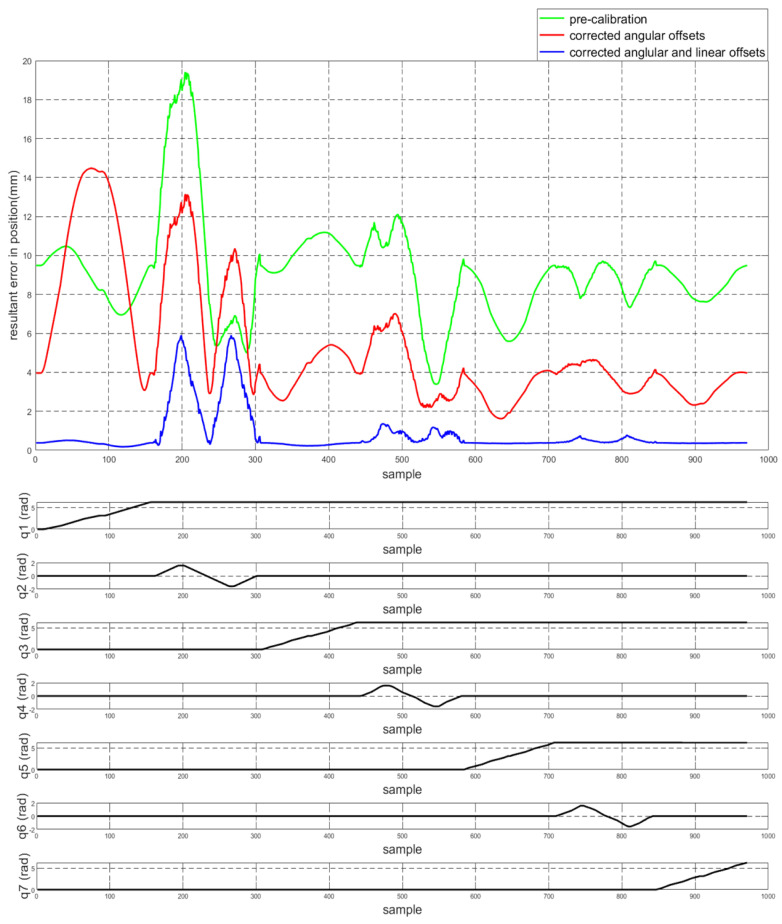
The plot for the resultant end-effector position error for the training set before and after calibration (**top**) and the motion of each of the robot joints during the training set generation (**bottom**).

**Figure 4:**
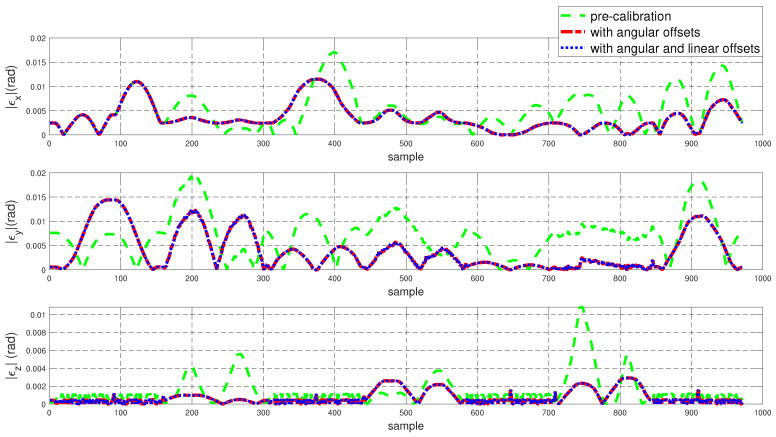
Error in end-effector orientation ${\left(R^{T}\tilde{R}-I\right)}^{\vee }$ for the training dataset before and after calibration (where R and $\tilde{R}$ are the end-effector rotation matrices obtained using the kinematic model and the feedback respectively).

To assess the performance of the calibrated model on the training dataset, both the position and orientation errors have been computed before (Equation (1)) and after (Equation (11)) the calibration. Figure 3 depicts the plots for the absolute position error without calibration ($\tilde{P}\left(q\right)-P_{p}\left(q\right)$), with angular offset calibration ($\tilde{P}\left(q\right)-P_{p}\left(q^{\prime }\right)$) and with both linear and angular calibration ($\tilde{P}\left(q\right)-P_{p^{\prime }}\left(q^{\prime }\right)$) for the end-effector. For better insight, the robot joint angle profile during the training data collection is also added. From the figure, a qualitative observation can be made that the calibration improves the resultant position accuracy. From Table 4, it can be observed that our calibrated model managed to bring down the maximum error by 69.59 % and the mean error by 91.29%. The orientation error ${\left(R^{T}\tilde{R}-I\right)}^{\vee }$ is computed and plotted in Figure 4. With reference to Table 5, by compensating for both the angular and linear offsets, the maximum error for each of the x, y and z dimensions ($|\epsilon_{x}\left|,\right|\epsilon_{y}|$ and $|\epsilon_{z}|$ respectively) are brought down by 32.75%, 25.77% and 73.15% whereas the mean errors came down by 31.37%, 44.29% and 53.3% respectively. It is evident that the orientation error does not change by introducing the linear offsets, which is in line with our assumption that the link length does not affect the orientation of the robot.

### 5.3. Validation on the Test Dataset

**Figure 5:**
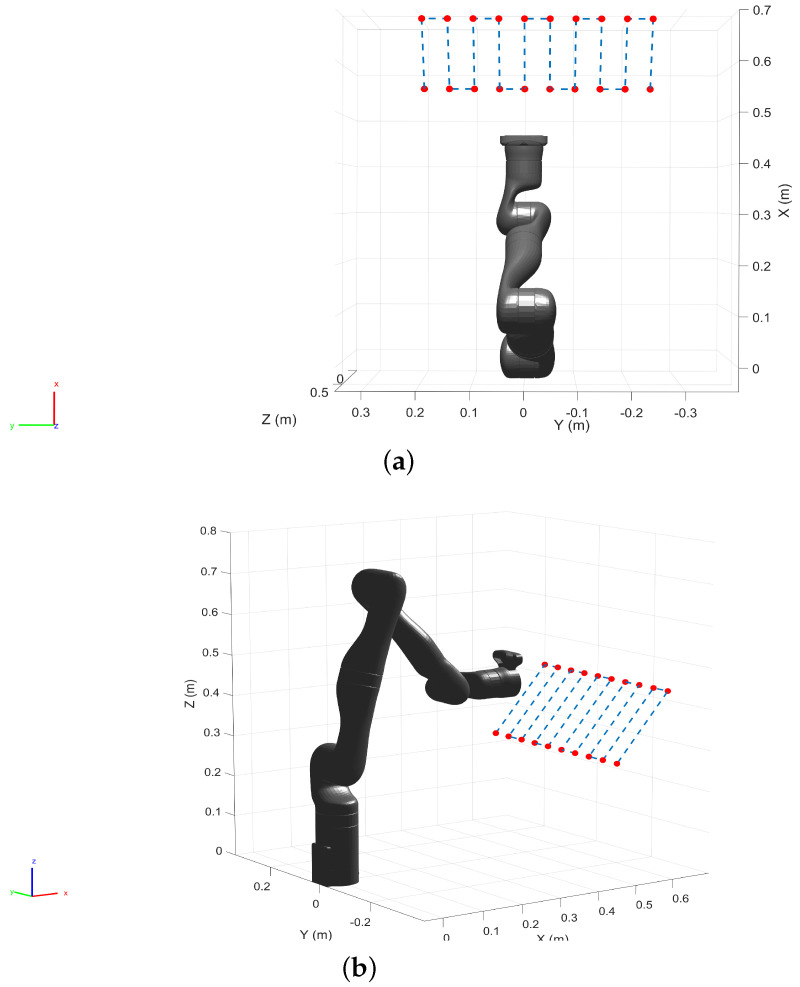
Spatial data points are generated to collect test dataset. While the robot moves to each of the locations with a raster motion, the position and the orientation of the end-effector along with the joint angle are logged. (**a**) Top view. (**b**) Perspective view.

To validate the proposed approach against a test set, we generated a set of spatial locations spanning the first two quadrants of the robot base frame (Figure 5). The robot was commanded to move to these location resulting in a raster motion in position control mode, starting from the left to the right while the end-effector position and orientation were logged.

**Figure 6:**
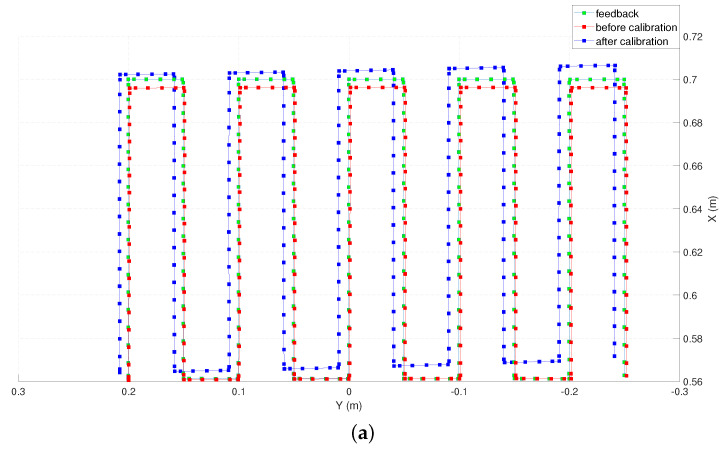

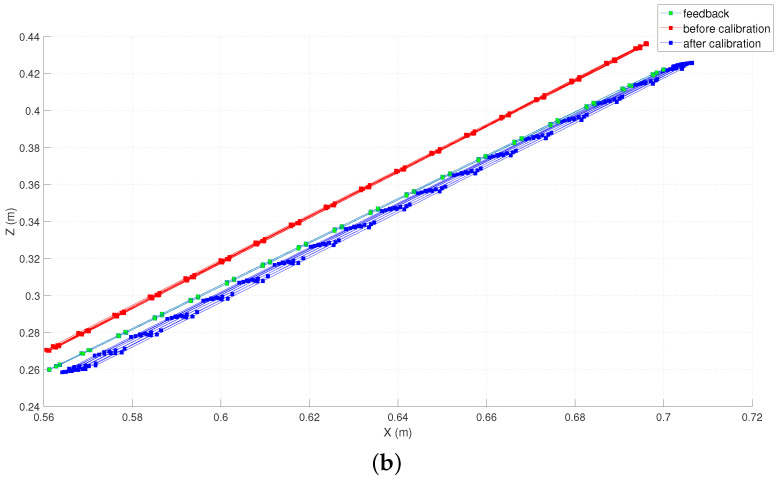
Estimated end-effector position before and after calibration (down-sampled by 5) plotted along with the feedback positions. (**a**) Top view (X-Y plane). (**b**) side view (X-Z plane).

We computed the trajectory with the un-calibrated and the calibrated models, which are superimposed on the commanded trajectory for comparison in Figure 6.

**Figure 7:**
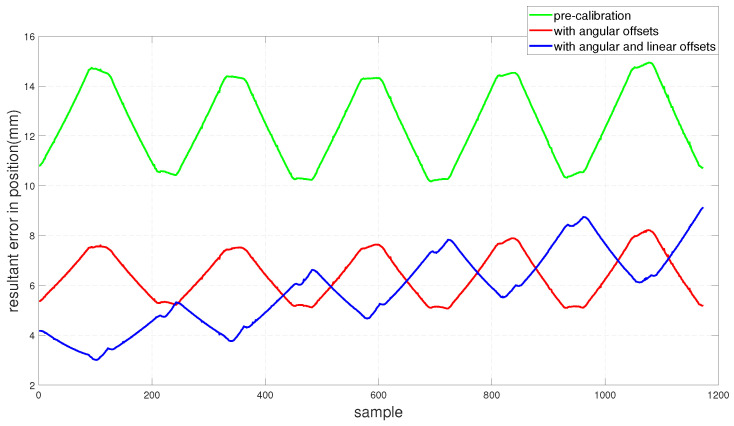
Resultant end-effector position error for the test set before and after calibration.

**Figure 8:**
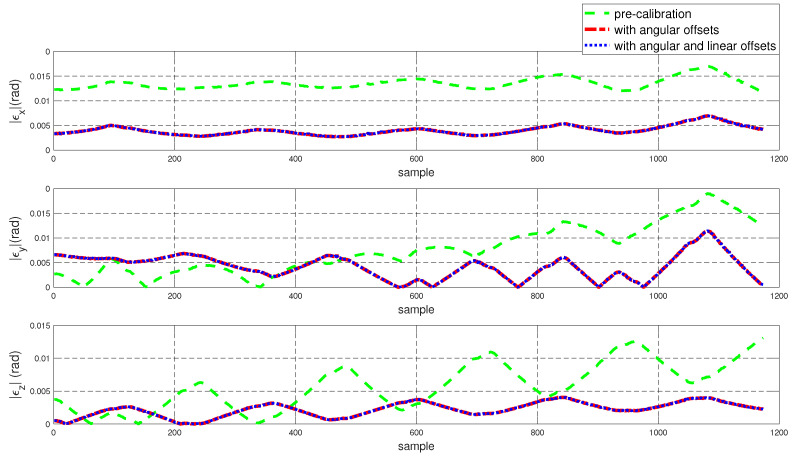
Error in end-effector orientation error ${\left(R^{T}\tilde{R}-I\right)}^{\vee }$ for the test dataset before and after calibration.

The position and orientation error plots before and after the calibration are shown in Figure 7 and Figure 8 respectively. Despite the fact that the test data was generated mostly from a different subspace of the workspace compared to the training dataset, the calibrated model managed to bring down the resultant position error as can be observed in Figure 7. By compensating for the angular offsets alone, the maximum and the mean position errors were brought down by 44.97% and 48.8% respectively. With reference to Table 6, by accommodating both the angular and the linear offsets, the maximum position error is reduced by 38.93% whereas the mean error came down by 53.6%. Table 7 shows that, by compensating for both the angular and linear offsets, the maximum orientation error for each of the x, y and z dimensions is brought down by 59.17%, 39.47% and 68.70% whereas the mean errors are lowered by 70.30%, 45.60% and 61.40% respectively.

**Figure 9:**
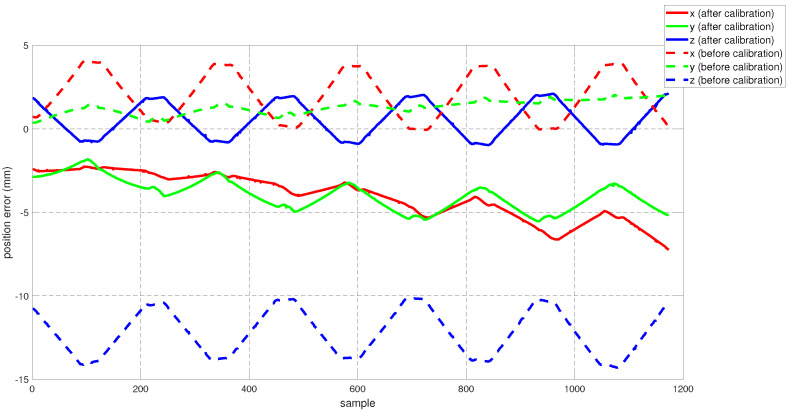
Test set position error along individual axes.

During the validation on the test set, while our approach was successful in bringing down the resultant kinematic error, the corrected model did not perform well in the X and Y axes individually (See Figure 9). However, often the X-Y accuracy is highly desirable in the context of planar tasks where the task space is simply the X-Y plane. We hypothesise the error in the X-Y plane is because of the fact that very few training data samples were collected from the desired subspace of the robot configuration space during the training set generation (See Figure 2). To validate the hypothesis, we devised a second experiment where we collected more training data samples from the desired subspace of the task space so that the calibrated model is more meaningful to the task to be performed by the robot.

## 6. Operation Space Targeted Calibration

**Figure 10:**
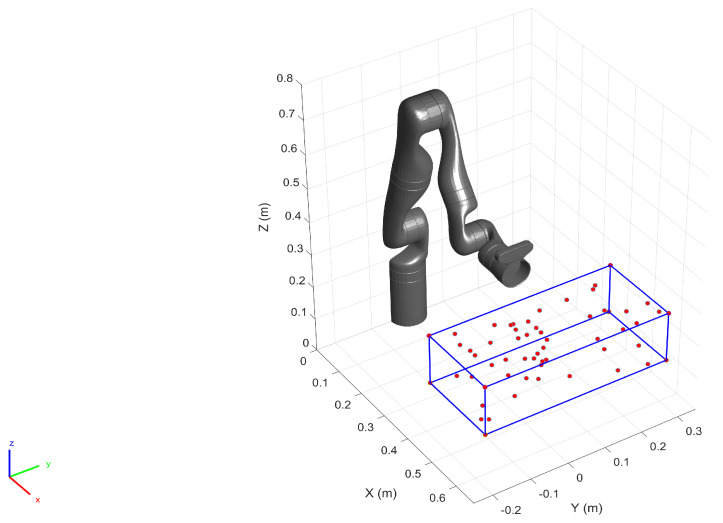
For localized calibration a work volume is defined (formed by the blue lines), within which 58 spatial points (red circles) were generated. The robot moves to each of the 3D points, and the simultaneous logging of the robot position and the orientation populate the training set.

In this section, we perform the calibration by collecting additional training data samples from the pre-defined operating space of the robot. To that end, we first defined a work volume for the robot within which we assume the robot performs a given task (See Figure 10). We also generated a total of 58 spatial locations within the work-volume (including the vertices) as the goal positions for the robot. In order to populate the training dataset we commanded the robot (in position control mode) to move to each of the generated goal positions while logging the position and the orientation of the end-effector along with the joint configuration. The robot was commanded to maintain a constant orientation throughout the motion. The additional training data samples together with the original data samples (See Figure 3) generate the final training dataset for calibration. Here, almost 25% of the training set is composed of the additional data samples.

The calibration preformed on the test dataset yielded a set of calibration parameters as tabulated in Table 8 and the corrected kinematic model is considered to be Model 2.

### 6.1. Validation on the Training Set

**Figure 11:**
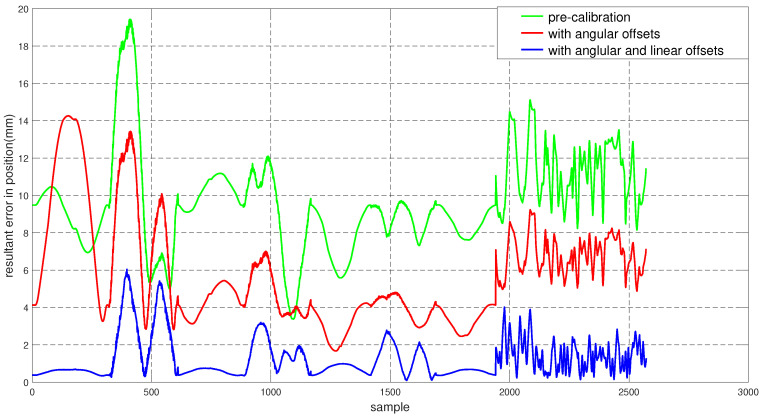
Resultant end-effector position error for the training set before and after calibration. The error corresponding to the additional training data starts at sample number 1940.

**Figure 12:**
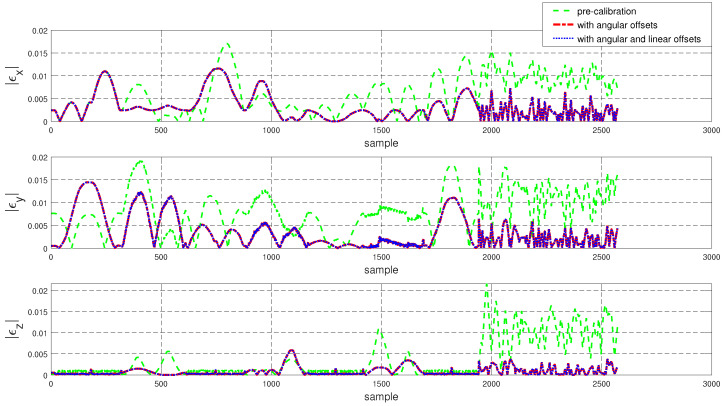
Error in end-effector orientation error ${\left(R^{T}\tilde{R}-I\right)}^{\vee }$ for the training dataset before and after calibration.

To evaluate the performance of Model 2 on the training set, both the position and the orientation errors have been computed and plotted in Figure 11 and Figure 12 respectively. The statistical analysis for the error data is carried out and outlined in Table 9 and Table 10.

### 6.2. Validation on the Test Set

**Figure 13:**
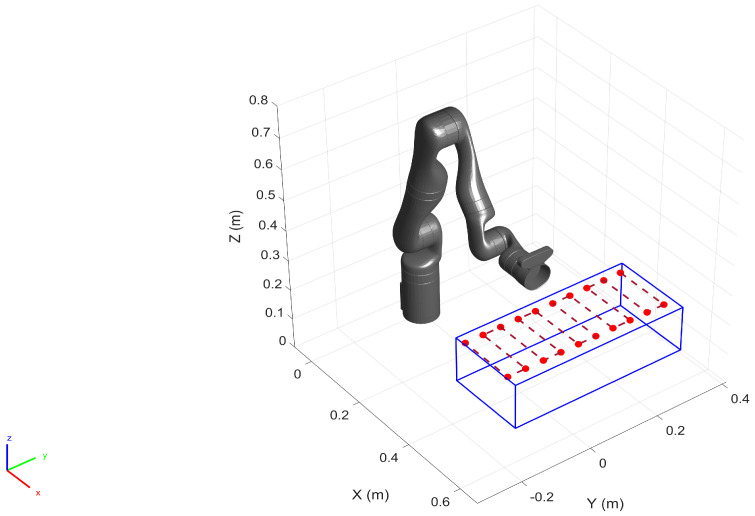
Spatial data points are created within the pre-defined work volume and the test data samples are collected as the robot moves to each of the locations.

To populate the test dataset, we generated 20 spatial points in the pre-defined work volume as shown in Figure 13. The robot (in position control mode) was commanded to move to each of these locations by performing a raster motion maintaining a constant orientation as illustrated in Figure 13. The Cartesian position and the orientation of the end-effector were logged together with the robot joint configuration.

**Figure 14:**
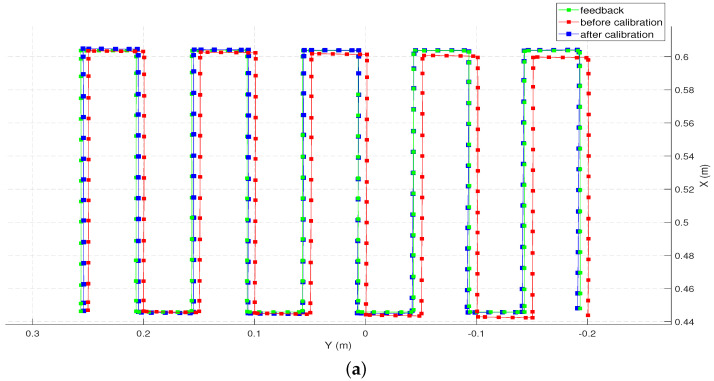

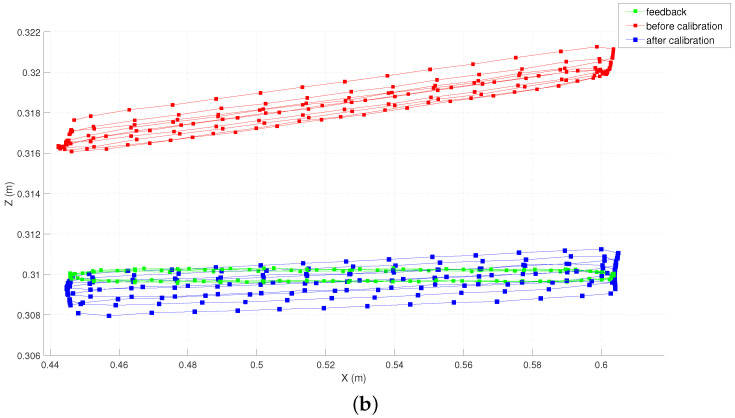
Estimated end-effector position before and after calibration (down-sampled by 10) plotted along with the feedback positions. (**a**) Top view (X-Y plane). (**b**) side view (X-Z plane).

To evaluate the accuracy along individual axes, the commanded trajectory for the robot, the factory calibrated feedback from the controller, the forward kinematics given by the calibrated and the un-calibrated models are plotted on the X-Y and the X-Z planes as shown in Figure 14 (The data points representing the position before and after calibration are down-sampled by 10 for clarity of illustration). Qualitatively, it can be observed that the end-effector position computed by the calibrated model outperforms the one computed by the un-calibrated model along all three axes.

**Figure 15:**
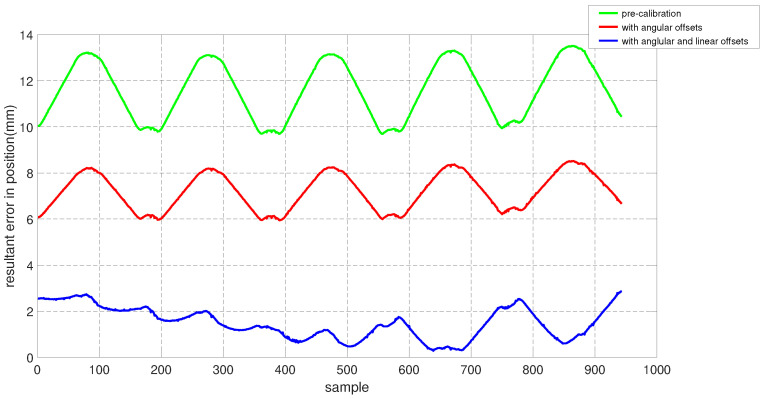
Resultant end-effector position error for the training set before and after calibration.

**Figure 16:**
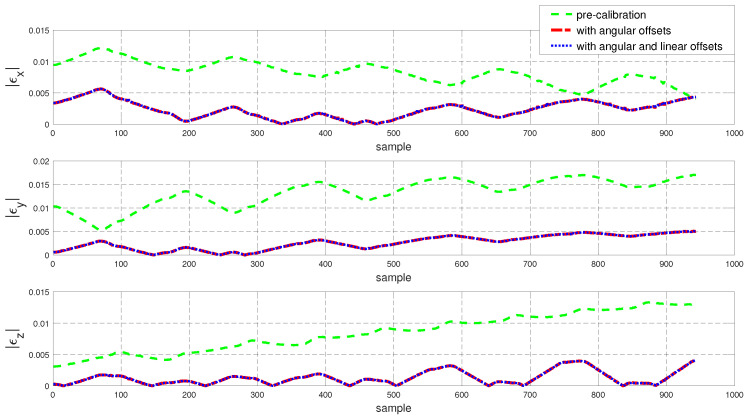
Resultant error in end-effector orientation error ${\left(R^{T}\tilde{R}-I\right)}^{\vee }$ for the training dataset before and after calibration.

The performance of the calibrated model on the test data is evaluated and both the position and the orientation error are plotted in Figure 15 and Figure 16 respectively. In addition, the statistical analysis was performed and tabulated in Table 11 and Table 12.

**Figure 17:**
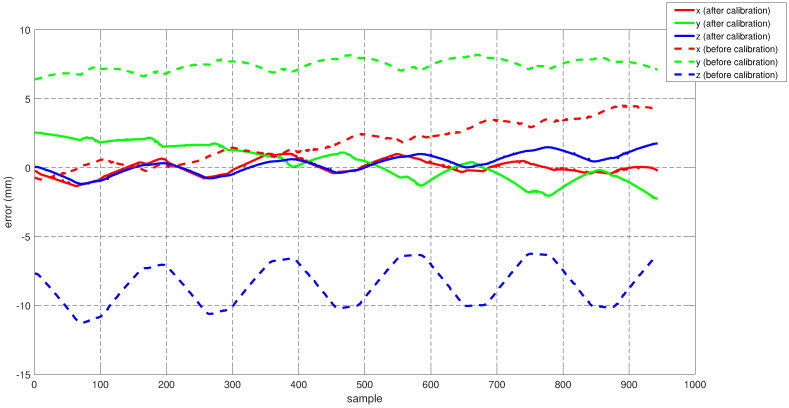
Test set position error along individual axes.

To perform a quantitative analysis, the error values for each of the axes are plotted in Figure 17. From the plot, it can be noticed that the calibrated model performs significantly better in comparison to its un-calibrated counterpart.

**Figure 18:**
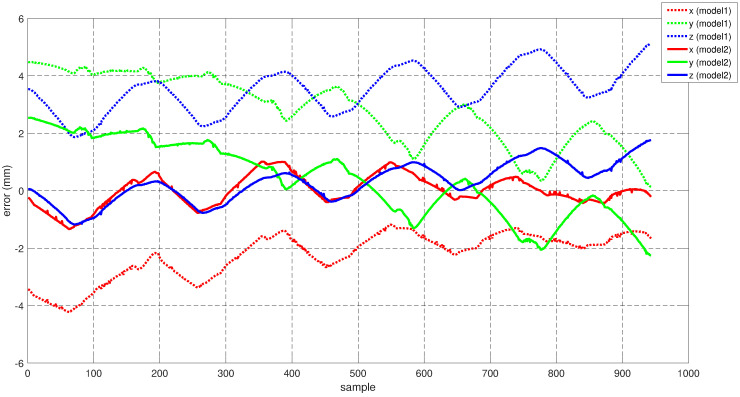
Comparison of the error along the coordinates axes for Model 1 and Model 2.

Now to validate our hypothesis regarding the error in the X-Y plane for Model 1, in Figure 18 we compare the error measurements along the X, Y and Z axes both for Model 1 and Model 2. It can be observed that for the same test dataset, Model 2 (i.e., obtained by collecting for training samples from the operating space of the robot) exhibits a better performance in comparison to Model 1. This aligns with our hypothesis that the increase in the X-Y errors in Section 5.3 (Figure 9) is due to the fact that very few training data samples were collected from the desired subspace of the robot configuration space.

## 7. Discussion

The work presented in this paper focuses on compensating for the linear and the angular offsets that adversely affect the position accuracy of the robot. However, we do not account for factors such as the compliance in the link or the joint transmission systems. The influence of these components comes into play significantly under the presence of a payload or even simply gravity itself, particularly under configurations which impose relatively higher torques on the links and joints. We observed that during the logging of the training dataset, the robot passes through a total of six such configurations (Figure 2b,d,f) at which higher gravitational torques are imposed due to the extension of the distal bodies. We can observe that corresponding to these configurations there are three pairs of identical peaks in the resulting error plots (see Figure 3) which diminish in magnitude as the robot motion progresses to the distal joints (also lower gravitational loading). Hence, we infer that the peaks with relatively larger values of errors are generated due to the link deflection under the increased gravitational loading, which was unaccounted for during the calibration.

## 8. Conclusions

Robot calibration is a necessity in planning and executing both contact and non-contact tasks alike, reliably and safely. This paper presents a fast re-calibration method to improve the robot position and orientation accuracy by compensating for the joint offset error as well as for the discrepancies in the link dimensions. Our approach is based on the assumption that the actual forward kinematics is known with sufficient accuracy, possibly through the factory calibrated feedback from the controller. A set of parameters to account for the linear and angular discrepancies are identified by minimising the mismatch between feedback and the computed forward kinematics. The proposed calibration approach brought about a significant improvement in the forward kinematics in comparison with an un-calibrated model, which is backed up by experimental analysis.

## Figures and Tables

**Table 1:** Brief comparison of kinematic calibration techniques.

Paper	Type of Calibration	FK Measurement Technique (for Ground Truth)	Calibration Method	Type of Regression	Application Scenarios	Final Position Error	Error Reduction %
Li et al., 2019 [37]	Open-loop	Leica Geosystems Absolute Tracker (AT960)	Dual quaternion-based calibration (DQBC) algorithm based on FK obtained from DH notation and; modified DQBC	Least-squares	An error model for serial robot kinematic calibration based on dual quaternions. Used to calibrate dual arm 7-DoF SDA5F robot.	Arm1: 0.4523 mm Arm2: 0.7109 mm	86.56 81.25
Wang et al., 2014 [6]	Open-loop	FARO arm to measure ball target position.	Product of Exponential (POE) and adjoint transformation based FK	Least-squares	Analytical approach to determine and eliminate the redundant model parameters in serial-robot kinematic calibration based on the POE formula	Max. error 2.2 mm	-
Li et al., 2016 [18]	Open-loop	FARO Laser Tracker ION	Product of Exponential (POE) for FK. Algorithm based on the ACS (axis configuration space) and adjoint error mode	Least-squares	Novel kinematic calibration algorithm based on the ACS and Adjoint error model. It is computationally efficient and can easily handle additional assumptions on joint axes relations.	Mean error SCARA: 0.07 mm Kawasaki: 0.063 mm Max. error SCARA: 0.16 Kawasaki: 1.23 mm	-
Liu et al., 2018 [19]	Open-loop	Leica Laser Tracker	Product of Exponential (POE)	Least-squares	Calibration of serial robot based on local POE formula for fastener hole drilling in aircraft assembly.	Mean error 0.144 mm Max. error 0.301 mm	- 97.30
Gharaaty et al., 2018 [28]	Open-loop	C-Track 780 from Creaform	Dynamic pose correction with PID controller	Root Mean Square (RMS)	Online pose correction of 6 DoF industrial robots, FANUC LR Mate 200iC and FANUC M20iA, using an optical CMM system for high accuracy applications such as riveting, drilling and spot welding.	Max. error 0.05 mm	79.17
Motta et al., 2016 [5]	Open-loop	ITG ROMER	Levenberg–Marquardt algorithm to solve non-linear least squares problem	Non-linear least-squares	Calibration optimization of a 5-DoF robot for repairing the surface profiles of hydraulic turbine blades.	Max. error 0.15 mm	-
Joubair et al., 2015 [31]	Closed-loop	Two-in datum spheres separated by precisely known distances measured on a CMM	Mathematical optimization	RMS error minimization	Geometric Calibration of a six-axis serial industrial robot, FANUC LR Mate 200iC in a specific target workspace using distance and sphere constraints.	Mean error 0.086 mm Max. error 0.127 mm	87.68 90.39
Lattanzi et al., 2020 [9]	Open-loop	FARO Vantage laser tracker	Levenberg-Marquardt mathematical optimization	Non-linear least squares solver	Geometric calibration of 6-axis, DENSO VS-087 and 7-DoF TIAGo robotic arms for high accuracy manufacturing task.	Mean error DENSO VS-087: 0.06 mm TIAGo: 1.08 mm Max. error DENSO VS-087: 0.1 mm TIAGo: 2.83 mm	- TIAG0: 91.91
Proposed Method: Fast kinematic re-calibration	Open-loop	Factory calibrated feedback from the robot controller (No additional equipment required)	Compensating for the joint and link length offsets to calibrate the ideal robot model	Least-squares	Quick kinematic re-calibration of Kinova Gen3 Ultralightweight 7-DoF robot arm by compensating for joint and link length offsets.	Mean error 1.47 mm Max. error 2.87 mm	87.15 78.77

**Table 2:** Homogeneous transformation matrices for Kinova Gen3 7DoF robot (provided by manufacturer).

Transformation (Frame *n* to n−1)	$R{\left(q_{n}\right)}_{n}^{n-1}$	$p_{n}^{n-1}\left(m\right)$
Frame 1 to Base frame	$\left[\begin{array}{ccc} cq_{1} & -sq_{1} & 0\\ -sq_{1} & -cq_{1} & 0\\ 0 & 0 & -1 \end{array}\right]$	$\left[\begin{array}{cc} & 0\\ & 0\\ & 0.1564 \end{array}\right]$
Frame 2 to Frame 1	$\left[\begin{array}{ccc} cq_{2} & -sq_{2} & 0\\ 0 & 0 & -1\\ sq_{2} & cq_{2} & 0 \end{array}\right]$	$\left[\begin{array}{cc} & 0\\ & 0.0054\\ & -0.1284 \end{array}\right]$
Frame 3 to Frame 2	$\left[\begin{array}{ccc} cq_{3} & -sq_{3} & 0\\ 0 & 0 & 1\\ -sq_{3} & -cq_{3} & 0 \end{array}\right]$	$\left[\begin{array}{cc} & 0\\ & -0.2104\\ & -0.0064 \end{array}\right]$
Frame 4 to Frame 3	$\left[\begin{array}{ccc} cq_{4} & -sq_{4} & 0\\ 0 & 0 & -1\\ sq_{4} & cq_{4} & 0 \end{array}\right]$	$\left[\begin{array}{cc} & 0\\ & -0.0064\\ & -0.2104 \end{array}\right]$
Frame 5 to Frame 4	$\left[\begin{array}{ccc} cq_{5} & -sq_{5} & 0\\ 0 & 0 & -1\\ -sq_{5} & -cq_{5} & 0 \end{array}\right]$	$\left[\begin{array}{cc} & 0\\ & -0.2084\\ & -0.0064 \end{array}\right]$
Frame 6 to Frame 5	$\left[\begin{array}{ccc} cq_{6} & -sq_{6} & 0\\ 0 & 0 & -1\\ sq_{6} & cq_{6} & 0 \end{array}\right]$	$\left[\begin{array}{cc} & 0\\ & 0\\ & -0.1059 \end{array}\right]$
Frame 7 to Frame 6	$\left[\begin{array}{ccc} cq_{7} & -sq_{7} & 0\\ 0 & 0 & 1\\ -sq_{7} & -cq_{7} & 0 \end{array}\right]$	$\left[\begin{array}{cc} & 0\\ & -0.1059\\ & 0 \end{array}\right]$
Frame 7 to end-effector frame	$\left[\begin{array}{ccc} 1 & 0 & 0\\ 0 & -1 & 0\\ 0 & 0 & -1 \end{array}\right]$	$\left[\begin{array}{cc} & 0\\ & 0\\ & -0.0615 \end{array}\right]$

**Table 3:** Calibration parameters for Model 1.

Joint Frame (*n*)	$\delta q_{n}$ (rad)	$\delta p_{n}$ (m)
Base frame	NA	NA
frame 1	0.0044	[0.0085, 0.0003, −0.0083]
frame 2	0.0088	[−0.0068, 0, 0]
frame 3	−0.0035	[−0.0001, −0.0041, 0.0028]
frame 4	−0.0043	[−0.0003, 0.0015, 0]
frame 5	0.0068	[0.0001, 0, 0]
frame 6	0.0026	[−0.0003, −0.0001, −0.0024]
frame 7	−0.0084	[0.0009, 0, 0]
End-effector frame	NA	[0.0009, −0.0003, 0]

**Table 4:** Resultant position error before and after calibration.

Error	Before Calibration (mm)	With Angular Offsets (mm)	% Reduction in Error with Angular Offsets	With Linear and Angular Offsets (mm)	% Reduction in Error with Angular Linear and Angular Offsets
Max error	19.4	14.5	25.26	5.9	69.59
Mean error	9.1 ± 2.7	5.3 ± 3.3	41.76	0.8 ± 1.1	91.29

**Table 5:** Resultant orientation error before and after calibration.

Error		Before Calibration ($\times 10^{-2}$ rad)	With Angular Offsets ($\times 10^{-2}$ rad)	With Linear and Angular Offsets ($\times 10^{-2}$ rad)	% Reduction in Error
$|\epsilon_{x}|$	Max error	1.71	1.15	1.15	32.75
	Mean error	0.51 ± 0.39	0.35 ± 0.28	0.35 ± 0.28	31.37
$|\epsilon_{y}|$	Max error	1.94	1.44	1.44	25.77
	Mean error	0.70 ± 0.43	0.39 ± 0.4	0.39 ± 0.4	44.29
$|\epsilon_{z}|$	Max error	1.08	0.29	0.29	73.15
	Mean error	0.15 ± 0.17	0.074 ± 0.075	0.07 ± 0.075	53.33

**Table 6:** Resultant position error before and after calibration.

Error	Before Calibration (mm)	With Angular Offsets (mm)	% Error Reduction with Angular Offsets	With Linear and Angular Offsets (mm)	% Error Reduction with Linear and Angular Offsets
Max error	14.9	8.2	44.97	9.1	38.93
Mean error	12.5 ± 1.5	6.4 ± 0.9	48.8	5.8 ± 1.5	53.6

**Table 7:** Resultant orientation error before and after calibration.

Error		Before Calibration ($\times 10^{-2}$ rad)	With Angular Offset ($\times 10^{-2}$ rad)	With Linear and Angular Offsets ($\times 10^{-2}$ rad)	% Reduction in the Calibration Error
$|\epsilon_{x}|$	Max error	1.69	0.69	0.69	59.17
	Mean error	1.35 ± 0.11	0.40 ± 0.93	0.40 ± 0.93	70.30
$|\epsilon_{y}|$	Max error	0.76	0.46	0.46	39.47
	Mean error	0.31 ± 0.2	0.17 ± 0.09	0.17 ± 0.09	45.60
$|\epsilon_{z}|$	Max error	1.31	0.41	0.41	68.70
	Mean error	0.57 ± 0.35	0.22 ± 0.11	0.22 ± 0.11	61.40

**Table 8:** Calibration parameters for Model 2.

Joint Frame (*n*)	$\delta q_{n}$ (rad)	$\delta p_{n}$ (m)
Base frame	NA	NA
frame 1	0.0048	[0.0082, 0.0003, −0.0078]
frame 2	0.0080	[−0.0063, −0.0029, 0]
frame 3	−0.0076	[−0.0000, 0, 0]
frame 4	−0.0034	[ 0.0000, 0, −0.0043]
frame 5	0.0110	[0.0001, −0.0020, −0.0017]
frame 6	0.0025	[−0.0023, −0.0000, 0]
frame 7	−0.0090	[0.0026, 0.0004, 0]
End-effector frame	NA	[ 0.0007, −0.0006, 0]

**Table 9:** Resultant position error before and after calibration.

Error	Before Calibration (mm)	With Angular Offsets (mm)	% Reduction in Error with Angular Offsets	With Linear and Angular Offsets (mm)	% Reduction in Error with Angular Linear and Angular Offsets
Max error	19.43	14.27	26.56	6.04	68.91
Mean error	9.66 ± 2.61	5.80 ± 2.88	39.95	1.26 ± 1.08	86.96

**Table 10:** Resultant orientation error before and after calibration.

Error		Before Calibration ($\times 10^{-2}$ rad)	With Angular Offsets ($\times 10^{-2}$ rad)	With Linear and Angular Offsets ($\times 10^{-2}$ rad)	% Reduction in Error
$|\epsilon_{x}|$	Max error	1.71	1.16	1.16	32.16
	Mean error	0.63 ± 0.41	0.31 ± 0.28	0.31 ± 0.28	50.79
$|\epsilon_{y}|$	Max error	1.94	1.45	1.45	25.26
	Mean error	0.81 ± 0.45	0.35 ± 0.37	0.35 ± 0.37	56.79
$|\epsilon_{z}|$	Max error	2.16	0.58	0.58	73.15
	Mean error	0.38 ± 0.46	0.09 ± 0.10	0.09 ± 0.10	76.32

**Table 11:** Resultant position error before and after calibration.

Error	Before Calibration (mm)	With Angular Offsets (mm)	% Reduction in Error with Angular Offsets	With Linear and Angular Offsets (mm)	% Reduction in Error with Angular Linear and Angular Offsets
Max error	13.52	8.52	36.98	2.87	78.77
Mean error	11.44 ± 1.21	7.08 ± 0.80	38.11	1.47 ± 0.66	87.15

**Table 12:** Resultant orientation error before and after calibration.

Error		Before Calibration ($\times 10^{-2}$ rad)	With Angular Offsets ($\times 10^{-2}$ rad)	With Linear and Angular Offsets ($\times 10^{-2}$ rad)	% Reduction in Error
$|\epsilon_{x}|$	Max error	1.22	0.56	0.56	54.10
	Mean error	0.83 ± 0.17	0.22 ± 0.13	0.22 ± 0.13	73.49
$|\epsilon_{y}|$	Max error	1.70	0.31	0.31	81.76
	Mean error	1.34 ± 0.28	0.26 ± 0.15	0.26 ± 0.15	80.60
$|\epsilon_{z}|$	Max error	1.34	0.41	0.41	69.40
	Mean error	0.83± 0.30	0.12 ± 0.10	0.12 ± 0.10	85.54

## Data Availability

Not applicable.
